# Seeking international consensus on approaches to primary tumour treatment in Ewing sarcoma

**DOI:** 10.1186/s13569-020-00144-6

**Published:** 2020-11-17

**Authors:** Craig Gerrand, Jessica Bate, Beatrice Seddon, Uta Dirksen, R. Lor Randall, Michiel van de Sande, Paul O’Donnell, John Tuckett, David Peake, Lee Jeys, Asif Saifuddin, Mel Grainger, Jeremy Whelan

**Affiliations:** 1 Royal National Orthopaedic Hospital, Brockley Hill, Stanmore, HA7 4LP Middlesex UK; 2 Southampton Children’s Hospital, University Hospital Southampton NHS Foundation Trust, Southampton, UK; 3 University College Hospital, 250 Euston Road, London, NW1 2PG UK; 4 Pediatrics III, Sarcoma Centre, West German Cancer Centre, German Cancer Consortium (DKTK), Center Essen, University Hospital Essen, University Duisburg, Hufelandstr. 55, 45122 Essen, Germany; 5 Department of Orthopaedic Surgery, UC Davis Health, 4860 Y Street, Suite 3800, Sacramento, CA 95817 USA; 6 Leiden University Medical Center, Albinusdreef 2, Leiden, The Netherlands; 7 Freeman Hospital, Newcastle-upon-Tyne, NE7 7DN UK; 8 Oncology-University Hospitals Birmingham NHS Foundation Trust, Birmingham, B15 2TH UK; 9 Royal Orthopaedic Hospital, Bristol Road South, Birmingham, B31 2AP UK; 10 University Hospital Birmingham, Edgbaston, Birmingham, B15 2GW UK

**Keywords:** Ewing sarcoma, Surgery, Radiotherapy, Limb salvage, Combined modality

## Abstract

**Background:**

The local treatment of Ewing sarcoma of bone involves surgery, radiotherapy or both. The selection of treatment depends on the anatomical extent of the tumour, the effectiveness of the proposed treatment, its morbidity, and the expectation of cure. However, not only are there variations in the approach to local treatment between individual patients, but also between treatment centres and countries. Our aim was to explore variation in practice and develop consensus statements about local treatment.

**Methods:**

A three stage modified Delphi technique was used with international collaborators. This involved an expert panel to identify areas of controversy, an online survey of international collaborators and a consensus meeting in London, UK in June 2017. In the consensus meeting, teams of clinicians discussed the local management of selected cases and their responses were collected with electronic voting.

**Results:**

Areas of greater or less consensus were identified. The lack of evidence underpinning different approaches was noted and areas for collaborative research became apparent.

**Conclusion:**

This has demonstrated that there is an international consensus around many aspects of the local treatment of Ewing sarcoma of bone, including the use of specialist MultiDisciplinary Team (MDT) meetings with access to all appropriate treatments. However, considerable variation remains including the use of different staging investigations, decision making, definitions of response, and radiotherapy doses and timing. Further collaborative work should be undertaken to determine the impact of these variations in order to define best practice.

## Background

Ewing sarcoma is the second most common primary bone tumour occurring in children and young adults, with an incidence of approximately 1 per million. It typically occurs in the second and third decades of life, with 80% of patients under 20 years of age at diagnosis [1]. Ewing sarcoma can occur in any bone, with a distribution reflecting the mass of bone in the skeleton [2].

Ewing sarcoma of bone classically presents with pain and an extra-osseous mass arising from the affected bone. Characteristic imaging features include a periosteal reaction with or without bone destruction on plain radiographs, and an extra-osseous mass on magnetic resonance imaging (MRI). Most cases demonstrate a translocation involving chromosomes 11 and 22 [3].

Treatment is multidisciplinary and highly specialised, involving chemotherapy, radiotherapy and surgery. Local therapy decisions are often nuanced, balancing the risks and morbidity of surgery and radiotherapy with potential benefits. Approaches to local treatment vary between countries, reflecting different health care structures and philosophies. In the EICESS 92 study, differences in local treatment approaches were identified: patients from the United Kingdom were less likely to receive combined modalities (surgery and radiotherapy) than those treated in Germany, and there were differences in local relapse and overall survival [4].

Recognising these differences, an international consensus meeting to discuss the local treatment of Ewing sarcoma was held in Birmingham in 2007. This resulted in several changes in UK practice, including a pilot in which a national Ewing Multidisciplinary Team panel (NEMDT) provided central review of cases. This process has been associated with changes in treatment approaches, in particular the greater use of combined modalities and especially of preoperative radiotherapy. In June 2017, a second consensus meeting was held in London to explore current differences in local treatment approaches and to inform the NEMDT panel. The specific objectives were to establish standards for current practice, develop consensus statements to guide local treatment decisions and identify areas for further research.

## Methods

A modified Delphi method was used comprising a three stage process of expert panel review, a pre-meeting online survey and case review with electronic voting at the meeting. Ewing sarcoma of bone arising in all anatomic sites other than the head and neck were included.^1^ Soft tissue Ewing sarcomas were excluded.

An expert scientific advisory board was convened in early 2017 to develop the content for the conference. This panel included clinicians from centres in the UK, Netherlands, Germany and the USA (CG, JB, JW, UD, LR, MvdS, DP, BS, LJ). Clinical situations in which the approach to treatment might vary were identified through discussion with the scientific advisory board and from case discussions at the UK NEMDT. A list of potential conference participants was compiled aiming to give a wide geographical representation, including centres recognised as having differing approaches to the treatment of Ewing sarcoma and participants in the previous consensus meeting.

Confirmed conference attendees were asked to complete a pre-meeting survey on the Slido platform (https://www.sli.do/) (Additional file 1, Appendix 1). The survey asked respondents about their specialty, facilities and services at their centre, and general approach to treatment.

The final phase was based around a multidisciplinary conference. The programme included introductory presentations summarising the principals of imaging and treatment at different anatomical sites. Clinical cases were then presented to the audience by two radiologists (P O’D and JT, Additional file 1, Appendix 2). Participants were asked about how they would treat each case, and differences in approach were openly discussed. At the end of the meeting, cases brought by attendees were presented to test the consensus. Participant responses were collected electronically using Slido (https://www.sli.do/).

### Levels of evidence and consensus

Using data from the pre-meeting survey and audience responses, consensus was evaluated as strong (more than 75% of participants/respondents agreeing with a statement), moderate (50–75% of participants/respondents agreeing with a statement) or none (no clear agreement). Response data are presented for each individual respondent, not by centre.

## Results

### Participants

#### Pre-meeting survey

The pre-meeting survey was sent to 81 clinicians who expressed an interest in attending, of whom 58 (72%) responded after two reminders. There were representatives from 27 centres in 15 countries, treating a median of 6–10 patients a year (range < 5 to > 50 cases per year).

Respondents were orthopaedic surgeons (16/58, 28%), paediatric oncologists (15/58, 26%), radiation oncologists (11/58, 19%), radiologists (6/58, 10%), medical oncologists (5/58, 9%), histopathologists (1/58, 2%), and other (4/58, 7%). The majority (48/58, 83%) had not attended the previous consensus meeting in Birmingham in 2007.

#### Consensus meeting attendees

There were 59 clinical voting attendees at the meeting. Thirty-four centres from 19 countries were represented. There were non-voting attendees from charities and research bodies. Voting attendees were orthopaedic surgeons (18/58, 32%), paediatric oncologists (17/58, 29%), radiation oncologists (9/58, 15%), radiologists (3/58, 5%), medical oncologists (8/58, 14%), histopathologists (1/58, 2%), and other (2/58, 4%).

### Patient pathways and services

#### The ideal pathway for patients

Survey respondents confirmed that specialist centres routinely perform conventional radiographs in 2 planes (Consensus level: strong; Q2.1a, 52/56, 93%) (summarised with other areas of consensus in Table 1), and MRI of the whole involved compartment with adjacent joints (Consensus level: strong; Q2.1b, 55/56, 98%). The majority perform staging computed tomography (CT) of the chest only (Consensus level: moderate; Q2.1d, 34/54, 63%) with a smaller proportion performing CT of the chest, abdomen and pelvis (Consensus level: none; Q2.1e, 18/45, 40%). Only a minority of respondents (Consensus level: none; Q2.1c, 13/55, 24%) were in centres routinely offering whole body MRI: more offered positron emission tomography (PET/CT) (Consensus level: moderate; Q2.1f, 37/53, 70%) and/or isotope bone scan (Consensus level: moderate; Q2.1g, 37/56, 66%) and bone marrow sampling (Consensus level: moderate; Q2.1h, 40/56, 71%), depending on the clinical scenario.

**Table 1:** Summary of consensus statements

Patient pathways and services
*Imaging at presentation should include*
Conventional X-rays in 2 planes (Strong)
MRI of the whole involved compartment and adjacent joints (Strong)
Staging CT of chest (Moderate)
PET/CT (Moderate)
Isotope bone scan (Moderate)
Bone marrow sampling (Moderate)
Patients should be managed within a properly constituted MDT (Strong)
*Services should have access to the following*
Whole body MRI (Strong)
Whole body CT/PET (Strong)
Specialist surgical teams (Strong)
Expert limb fitting/prosthetic services (Strong)
Specialist sarcoma rehabilitation (Strong)
Clinical nurse specialist support (Strong)
Clinical trials (Strong)
Radiotherapy by IMRT (Strong)
Radiotherapy by proton beam (Strong)
*Timing and approaches to decisions about local treatment*
Patients should have the opportunity to explore local treatment options as soon after diagnosis as possible (Strong)
Decisions about local therapy should be made in collaboration with patients and families (Strong)
It is possible to make a decision about radiotherapy based on the imaging at presentation in some situations (Moderate)
The radiological response to chemotherapy is important when considering local therapy options (Strong)
With widespread bone metastases, radiotherapy alone to the primary tumour is routinely indicated (Strong)
With oligometastases, radiotherapy alone may be considered as well as treatment to the oligometastases (Strong)
Patients with pulmonary metastases should be considered for the same local treatment as those without (Strong), including potentially morbid resections (Moderate)
*Pathology and molecular biology*
Patients should have biopsies in the bone cancer centre (Strong)
Core needle biopsies or open biopsies are preferred (Strong)
Specimens should be tested for cytogenetic abnormalities (Strong)
Oligometastases in lymph nodes or bone should be biopsied (Moderate)
Tissue is banked for research (Strong)
Assessment of histological response is important when considering the effectiveness of local treatment (Strong)
An adequate response to chemotherapy should be taken as > 90% necrosis (Moderate)
Surgical margin status is a reliable indicator of tumour left in the patient (Moderate)
An adequate surgical margin is one in which there is no viable tumour at the edge of the resection specimen (Moderate)
*Surgery*
The surgical resection should be planned to include the biopsy track (Strong)
An adequate surgical margin is one in which all of the anatomical structures involved at presentation are completely removed (Strong)
Where feasible it is reasonable to consider resection of peri-lesional oedema (Moderate)
The radiological response to neoadjuvant chemotherapy should be considered when planning surgery (Strong)
Pelvic spacers may have a role in reducing the morbidity of radiotherapy (Moderate)
Radiotherapy has a negative impact on outcomes after endoprosthetic replacement (moderate)
Radiotherapy has an negative impact on outcomes after allograft reconstruction (Moderate)
Radiotherapy does not make surgery more difficult technically (Moderate)
There is no role for debulking surgery when a tumour cannot be completely resected (Strong)
Local recurrence has an impact on overall survival (Strong)
*Anatomical site variations*
*Pelvis and sacrum*
Tumours which cross the midline in the sacrum are not considered resectable because of the morbidity associated with surgery (Strong)
Tumours with major visceral involvement or requiring pelvic organ removal may also be considered too morbid to resect (Moderate)
Definitive radiotherapy is indicated for unresectable sacral tumours (Strong)
Protons may be advantageous in the sacrum (Strong)
Preoperative radiotherapy may be preferred when the tumour volume is large (Moderate)
Radiotherapy is likely to be associated with increased complication rates (Strong)
*Spine*
Protons may be of some benefit in the spine (Strong)
The type of spinal reconstruction can affect the choice of radiotherapy treatment modality (Strong)
Patients with a possible Ewing’s tumour of the spine without neurological signs should have a biopsy before decompressive surgery (Strong)
Urgent surgery is recommended if there is a Ewing’s tumour of the spine causing neurological compromise (Moderate)
Radiotherapy is usually indicated after decompressive surgery (Strong) and should include the original tumour volume and all areas potentially contaminated by surgery (Strong)
*Chest*
A pleural effusion in relation to a chest wall tumour is not a definite indication for radiotherapy preoperatively (Moderate)
A pleural effusion in relation to a chest wall tumour may be an indication for post operative radiotherapy (Moderate)
Pleural involvement with a primary tumour may be an indication for preoperative (None) or postoperative (Moderate) radiotherapy
*Extremity*
Amputation is considered less often than for osteosarcoma (Strong)
Amputation may be indicated if negative margins cannot otherwise be achieved (Moderate)
If resection of a distal leg tumour would lead to inadequate margins or a foot with poor function, below knee amputation is indicated (Strong)
Amputation is less often recommended in the upper extremity (Moderate)
In the proximal tibia, amputation does not necessarily lead to better outcomes than proximal tibial replacement and radiotherapy (Moderate)
Radiotherapy can be added to surgery in the tibia but accepting a high risk of local complications (Moderate), therefore preoperative radiotherapy may be preferred (Moderate)
*Local therapy in advanced disease*
Suspected solitary bone metastases should be biopsied at presentation if possible (Strong)
Solitary bone metastases may be treated by surgery, radiotherapy or both if the morbidity is acceptable (Strong)
If there are widespread bone metastases, radiotherapy is indicated when symptomatic (Strong)
Potentially involved lymph nodes should have sampling or biopsy before chemotherapy if possible (Strong)
It is appropriate to surgically resect lymph nodes if there is suspicion of tumour involvement (Moderate)
It is reasonable to consider radical surgery such as amputation or hemipelvectomy to treat locally recurrent disease if there are no metastases (Strong)

#### The multidisciplinary team

Almost all survey respondents routinely discussed patients in a local MDT meeting (Consensus level: strong; Q3.1, 51/56, 91%), and a smaller number in a national MDT (Consensus level: none; Q3.1, 20/56, 36%) or routinely with teams in other centres (Consensus level: none; Q3.1, 7/56, 13%). In one centre patients were only discussed selectively outside it, and in an example of good practice, another described how the paediatric oncologist, surgeon and radiation oncologist held a joint consultation with all patients with pelvic and axial tumours at the same clinic visit after diagnosis and again at the time of a local control decision.

There is variation in guidelines for standard practice, as respondents reported adopting the EUROEWING 2012 trial protocol (Q3.2, 28/51, 55%), ESMO Clinical Practice Guidelines (20/51, 39%), British Sarcoma Group guidelines (14/51, 27%) and National Cancer Institute treatment guidelines (4/51, 8%). Other adopted protocols included those of the Childrens Oncology Group (COG), Scandinavian Sarcoma group, Ewing 2008, the French Combinair trial, EW-1 and EW-2 joint studies of Italian Paediatric Oncology Group (AEIOP) and the Italian Sarcoma group, and the hospital’s own guidelines (e.g. those of the Tata Memorial Hospital, Mumbai [5]).

#### Access to diagnostic and treatment services

The majority of respondents had access to whole body MRI (Consensus level: strong; Q 3.3, 42/56, 75%), whole body PET/CT (Consensus level: strong; 54/56, 96%), specialist surgical teams with sarcoma expertise (Consensus level; strong; 54/56, 96%), expert limb fitting/prosthetic services (Consensus level; strong; 52/56, 93%), specialist sarcoma rehabilitation (Consensus level; strong; 46/56, 82%), clinical nurse specialist support (Consensus level; strong; 44/56, 79%), clinical trials in Ewing sarcoma (Consensus level; strong; 50/56, 89%), and radiotherapy delivered by intensity modulated radiotherapy (IMRT) (Consensus level; strong; 52/56, 93%). Proton beam therapy was available (Consensus level; strong) in their centre (7/56, 13%), elsewhere in the country (20/56, 36%), or abroad (30/56, 54%). Most had access to specialist end of life/palliative care support (Consensus level; strong; 47/56, 84%).

Most surgeons have access to customised endoprostheses (4.4, 17/19, 89%), modular endoprostheses (18/19, 95%), “growing” endoprostheses (18/19, 95%), free flaps/vascularised grafts (18/19, 95%), rotationplasty (17/19, 89%), massive allografts (12/19, 63%), and extracorporeal irradiation/reimplantation (13/19, 68%).

### Timing and approaches to decisions about local treatment

#### Shared decision making

It was agreed that patients should have the opportunity to discuss local treatment options as soon after diagnosis as possible (Consensus level: strong; Q4.5o, 15/19, 79% agree/strongly agree). Decisions about local treatment should be made in collaboration with patients and families (Consensus level: strong; Q4.5p, 17/17, 100% agree/strongly agree).

There was moderate consensus that it is sometimes possible to make a decision about radiotherapy based on the imaging at presentation (Consensus level: moderate; Q5.6c, 14/26, 54% agree/strongly agree) and strong consensus that the radiological response to chemotherapy is important when considering local therapy options (Consensus level: strong, Q3.5a, 50/55, 91% strongly agree/agree).

#### Decisions about local treatment in the presence of metastatic disease

The statement that patients with bone metastases should have the same local treatment as those without did not reach consensus (Q3.5e, Agree/Strongly agree 30%, Undecided 26%, Disagree/strongly disagree 45%). With widespread bone metastases, radiotherapy alone to the primary tumour is routinely indicated (Consensus level: strong; Case 1. 44/54, 81% radiotherapy only to the primary tumour, also Case 3, 39/56, 70%, Case 17, 26/42, 62%). In the presence of oligometastatic bone disease, it may be reasonable to consider radiotherapy alone for the primary tumour (Consensus level: strong; Case5, 35/48, 73%) as well as radiotherapy to the metastases.

In contrast, patients with pulmonary metastases should be considered for the same local treatment as those without (Consensus level: strong; Q3.5f, 34/52, 65% agree/strongly agree). It is reasonable therefore to consider potentially morbid resections, for example of the pelvis, in the presence of pulmonary metastases (Consensus level: moderate; Case 2, 31/48, 65%).

### Pathology and molecular biology

#### Biopsy techniques and approaches

Patients usually have biopsies in the bone cancer centre (Q2.2, Consensus level: strong), with a core needle biopsy by a radiologist (Q2.3, 26/55, 47%), surgeon (14/55, 25%), or open incisional biopsy by a surgeon (12/55, 22%). Fine needle aspirate was only infrequently used (2/55, 4%). Biopsy tracks are usually marked so that they can be excised at the time of definitive surgery (Q2.4, 38/56, 68%).

Biopsy specimens are routinely tested for molecular abnormalities, including the EWS translocation (Q2.5, 53/56, 95%). Lymph nodes that may be involved on imaging should be sampled before chemotherapy (Q2.6, Consensus level: moderate, 28/56, 50%). Similarly, suspected bone oligometastases should be biopsied (Q2.7, Consensus level: moderate, 29/56, 52%). Tissue should be routinely banked for research (Consensus level: strong; Q2.8, 44/56, 79%).

#### Assessment of response to chemotherapy

The histological response to chemotherapy is important when considering the effectiveness of local treatment (Consensus level: strong, Q3.5b, 50/54, 92% strongly agree/agree). Some respondents agreed the definition of an adequate response to chemotherapy should be taken as > 90% necrosis (Consensus level: moderate. Q3.5c, 20/31, 64% strongly agree/agree), with others preferring 100% necrosis (Consensus level: weak. Q3.5d, 8/32, 25% strongly agree/agree). However, it was recognised that the increasing use of preoperative radiotherapy will change the interpretation of necrosis in resection specimens.

#### Assessment of surgical margins

Surgical margin assessment is a reliable predictor of tumour remaining in the patient (Consensus level: moderate; Q3.5g, 34/52, 66% strongly agree/agree). An adequate surgical margin is one in which there is no viable tumour at the edge of the resection specimen (Consensus level: moderate; Q35h, 33/53, 63% strongly agree/agree). The statement that an adequate surgical margin is one in which all of the anatomical structures involved before chemotherapy have been completely removed did not reach consensus in the pre-meeting survey (Q3.5i, 17/47, 37% Strongly agree/agree, 16/47, 35%, strongly disagree/disagree). There was strong consensus that local recurrence at the primary site has an impact on overall survival (Consensus level: strong; Q4.5f, 19/20, 95% agree/strongly agree).

### Surgery

#### Principles of surgery for Ewing sarcoma

When planning surgical resection the biopsy track should be removed (Consensus level: strong, Q4.1, 17/19, 89% “yes”).

In the pre-meeting survey, many respondents did not consider resecting all of the volume/anatomical structures involved before chemotherapy (Consensus level: moderate; Q4.2, 10/19, 53% “no”). However, most surgeons considered resecting all of the volume/anatomical structures involved after chemotherapy (Consensus level: strong; Q4.3, 16/19, 84% “yes”).

Where feasible it is reasonable to consider resection of peri-lesional oedema which might contain tumour (Consensus level: moderate; Case 4, 32/47, 68%),

The radiological response to neoadjuvant chemotherapy should be considered when planning surgery (Consensus level: strong; Q4.5n, 17/20, 85% agree/strongly agree).

Pelvic spacers should be considered to reduce the morbidity of radiotherapy (Consensus level: moderate; Q4.5j, 11/20, 55% agree/strongly agree).

#### Complications of surgery

Radiotherapy has a negative impact on outcomes, especially infection and prosthetic failure, after endoprosthetic replacement of a long bone (Consensus level: moderate; Q4.5b, 15/21, 71%), and after allograft reconstruction (Consensus level: moderate; Q4.5c, 15/21, 71%). Respondents thought radiotherapy did not make surgery more difficult technically (Consensus level: moderate; Q4.5d, 12/21, 57%).

Surgeons did not reach consensus about the statement that some patients at high risk of surgical complications should complete chemotherapy before surgical treatment (Consensus level: none; Q4.5a, 10/21, 48% strongly agree/agree vs 29% disagree/strongly disagree).

#### When is a tumour inoperable?

There is no role for debulking surgery when a tumour cannot predictably be completely resected (Consensus level: strong; Q4.5e, 18/20, 80% disagree/strongly disagree that debulking surgery should be considered).

### Radiotherapy

#### Indications for and timing of radiotherapy

Radiotherapy may be given pre-operatively, post-operatively or as definitive treatment. Tumour volume may be considered as a relative indication when making a recommendation for radiotherapy (Consensus level: none; Q5.4d, Q83).

There was no consensus as to whether pathological fracture at presentation was a definite indication for preoperative radiotherapy (Q75, with radiotherapy recommended always/very often in 29%, and rarely/never in 48%), although there was some agreement that radiotherapy should be considered postoperatively (Consensus level: moderate; Q87, 11/21, 53% Very often/always, compared with 24% rarely/never).

There was agreement that involved lymph nodes should be included in radiotherapy treatment volumes (Consensus level: moderate; Q91, 19/26, 73% agree/strongly agree).

If complete resection is possible based on initial imaging, the decision about radiotherapy can await assessment of histological response (Consensus level: strong; Case 11, 43/56, 77%).

A poor radiological response to neoadjuvant chemotherapy is a relative indication for preoperative (Consensus level: moderate; Q77, 61% sometimes/very often/always. Consensus level: strong; Case 9, 47/50, 94%) or postoperative radiotherapy (Consensus level: strong; Q89, 20/23, 87%, sometimes/very often/always).

Preoperative radiotherapy may be given when an inadequate (marginal) margin is anticipated on imaging. There was strong consensus that a 2 mm margin after resection and preoperative radiotherapy does not require further radiotherapy (Consensus level: strong, Case 8, 35/46, 76%). Tumours close to critical anatomical structures which would be morbid to resect surgically (e.g. major nerve or blood vessel) should be considered for preoperative radiotherapy (Consensus level: moderate; Q68, 12/24, 50% always/very often). The expectation of a close or positive surgical margin on the pre-chemotherapy scan is not a definite indication for preoperative radiotherapy (Consensus level: none; Q69). However, if a close or positive surgical margin is expected based on the post chemotherapy scan, preoperative radiotherapy should be considered (Consensus level: moderate; Q70, 14/24, 59%).

Indications for postoperative radiotherapy include viable tumour at the surgical margin (Consensus level: strong; Q79, 25/25, 100%, Very often/always), incomplete excision of the pre-chemotherapy tumour volume (Consensus level: moderate; Q80, 15/25, 60% very often/always) and poor histological response (Consensus level: moderate; Q81, 17/25, 68% very often/always), usually defined as < 90% necrosis, although there was variation in the level of histological response deemed acceptable to avoid radiotherapy (Consensus level: none; Case 9).

Complete excision and good histological response are needed to avoid radiotherapy. It is reasonable to add postoperative radiotherapy with a margin of < 2 mm and < 90% necrosis (Consensus level: strong; Case 7, 46/56, 82%).

#### Radiotherapy dose

For pre-operative radiotherapy, there was no consensus for dose (range 45–55.8 Gy), with doses of 45 Gy (27%), 50 Gy (32%), and a range of ‘other’ doses (41%) reported, including 50.4 Gy, 54 Gy and 55.8 Gy.

For post-operative radiotherapy, there was also no consensus, although the doses used were generally higher than for pre-operative radiotherapy (range 45–60 Gy). Doses reported were 45 Gy (18%), 50 Gy (23%), ‘other’ (50%), and ≥ 60 Gy (10%). The ‘other’ group included 42 Gy, 50.4 Gy, 54 Gy, and 55.8 Gy.

For definitive radiotherapy, there was again a lack of consensus, although as with post-operative radiotherapy, doses were generally higher than for pre-operative radiotherapy (range 45–70.2 Gy). Doses reported were 50 Gy (11%), 60 Gy (32%), > 60 Gy (11%) and ‘other’ (47%). The ‘other’ group included 45 Gy, 54 Gy, 55.8 Gy, 59.4 Gy, 70.2 Gy.

For all radiotherapy indications, fraction sizes of 1.5 Gy, 1.8 Gy and 2 Gy were reported.

#### Selection of modalities for planning and delivery of radiotherapy

There is an expectation that the indications for proton beam treatment will expand as it becomes more available (Consensus: moderate; Q92, 16/24, 67% agree/strongly agree).

Insertion of a pelvic spacer can be considered to allow delivery of the prescribed radiotherapy dose by reducing the dose to normal tissue structures, in particular bowel (Consensus level: moderate; Q51, 11/20, 55% agree/strongly agree).

### Anatomical site considerations

#### Pelvis/sacrum

There was strong consensus that sacral tumours which cross the midline should not be resected because the morbidity of resection including loss of bladder, bowel and sexual function is thought unacceptable (Consensus level: strong; Q4.6, 15/20, 75%. Also Case 15, 36/46, 78%). Similarly, major visceral involvement requiring pelvic organ removal would mean that most surgeons would not recommend resection (Consensus level: moderate; Q4.6, 14/20, 70%, Case 5). Definitive radiotherapy is indicated for sacral tumours for which surgery is considered too morbid (Consensus level: strong; Case 15, 44/44, 100%). Protons may be advantageous in this situation in terms of delivery of optimal dose, and reduction in normal tissue toxicity (Consensus level: strong; Case 15, 38/47, 81%).

Tumours in the pelvis may be selected for radiotherapy preoperatively (Consensus level: moderate; Q5.4h, 73% sometimes/very often/always) or postoperatively (Consensus level: strong; Q5.5h, 20/22, 91% sometimes/very often/always).

Preoperative radiotherapy may be preferred when the tumour volume is large (Consensus level: moderate; Case 2, 26/50, 52%; Case 10, 40/49, 82%; Consensus level: strong, Case 10, 39/52, 75%).

Reconstruction is likely to be associated with more complications if radiotherapy has been given and influences the choices for reconstruction (Consensus level: strong, Case 10, 43/49, 88%).

#### Spine

Proton beam radiotherapy may be of some benefit in the spine (Consensus level: strong; Case 12, 41/44, 93%: Case 13, 44/50, 88%). Where combined modality treatment with surgery and radiotherapy is indicated, it is recognised that the type of reconstruction can affect the choice of radiotherapy treatment modality (Consensus level: strong; Case 13, 32/43, 74%).

Patients who present with a possible Ewing tumour of the spine without neurological signs should have a biopsy as a response to systemic treatment is likely and may allow intralesional decompressive surgery to be avoided (Consensus level: strong; Case 14; 49/50, 98%).

If there is deteriorating or objective neurological compromise, urgent surgery is recommended (Consensus level: moderate; 29/48, 60%), rather than biopsy and chemotherapy/radiotherapy. Radiotherapy is invariably indicated after decompressive surgery (Consensus level: strong; Case 14, 41/46, 89%), and should include the original tumour volume and all areas potentially contaminated by surgery (Consensus level: strong; Case 14, 45/51, 88%).

There was no consensus about the use of craniospinal radiotherapy for patients with epidural disease (Q5.6d).

#### Chest

A pleural effusion in association with a chest wall tumour is not a definite indication for radiotherapy preoperatively (Consensus level: moderate; Q5.4e, 12/21, 57% rarely/never), but may be postoperatively (Consensus level: moderate; Q5.5e, 10/20, 50% always/very often). However, pleural involvement with a primary tumour may be an indication for radiotherapy either preoperatively (Consensus level: none; Q5.4f, 7/21, 34% very often/always) or postoperatively (Consensus level: moderate; Q5.5f, 10/20, 50% very often/sometimes. See also Case 17).

#### Extremity

Amputation is considered less often than for other extremity bone tumours (Consensus level: strong; Q4.5l, 16/20, 80%), but may be considered if negative surgical margins cannot otherwise be achieved (Consensus level: moderate; Q4.7, 14/20, 70%). Alternatively, if resection of a tumour would be associated with inadequate margins or a foot with poor function, below knee amputation may be indicated (consensus level: strong; Case 6. 43/54, 80%).

Respondents indicated that amputation would be less often recommended in an upper extremity tumour (Consensus level: moderate; Q4.5l, 10/20, 50%).

In proximal tibial tumours, it was agreed that amputation does not necessarily lead to better outcomes than proximal tibial replacement and radiotherapy (Consensus level: moderate; Q4.5k, 10/20, 50%). In the tibia, it is reasonable to add radiotherapy to surgery while accepting a high risk of local complications (Consensus: moderate; Case 3, 30/51, 59%). In this situation, preoperative radiotherapy is preferred (Consensus level: moderate; Case 3, 36/49, 73%). Preferred reconstructive options would be autograft, endoprostheses or allograft (Case 3). Amputation is a reasonable alternative for some patients.

In the proximal femur, if complete resection is deemed feasible, then either pre or post op radiotherapy can be considered. (Case 11).

In a case with an intraarticular tumour at the elbow, radiotherapy was recommended (Consensus level: strong; Case 18, 34/47, 72%), preferably postoperatively (Consensus level: moderate; Case 18, 31/50, 62%).

### Local therapy in advanced disease

#### General approach to patients with advanced disease

##### Oligometastases in bone

A suspected solitary bone metastasis should be biopsied at presentation if possible (Consensus level: strong; Case 4, 45/51, 88%). If there is more than one suspected metastasis it may be reasonable to biopsy more than one (Consensus level: moderate; Case 5. 32/56, 57%).

The statement that resection of bone metastases improves survival was not supported (Consensus level: none; Q4.5h), but bone metastases may be resected if the morbidity is acceptable (Consensus level: strong; Q4.5i, 16/20, 80% agree/strongly agree). A solitary metastasis should be treated by surgery, radiotherapy or both (Consensus level: strong; Case 4, 47/52, 90%, resection, radiotherapy or both). With greater numbers of bone metastases, individual treatment of all metastases becomes more difficult, but if possible radiotherapy to two lesions is reasonable (Consensus level: strong; Case 5, 49/51, 96%).

Where widespread bone metastases are present, radiotherapy is indicated when symptomatic (Consensus level: strong: Case 1, 50/57, 88%).

##### Lymph node involvement

Staging by PET/CT is more likely to detect soft tissue/lymph node involvement. Potentially involved lymph nodes should have sampling or biopsy before chemotherapy whenever possible (Consensus level: strong; Case 6; 44/50, 88%). It is appropriate to surgically resect lymph nodes if there is suspicion of tumour involvement (Consensus level: moderate; Q4.5g, 14/20, 70%. Also Case 6, consensus level: moderate; 31/56, 56%).

### Local therapy in relapsed disease

It is reasonable to consider radical surgery such as amputation or hemipelvectomy to treat locally recurrent Ewing sarcoma if there are no metastases (Consensus level: strong; Q4.5m, 18/20, 90% agree/strongly agree. Case 16, 31/48, 65%).

## Discussion

Our aim was to define existing international consensus about the local treatment of Ewing sarcoma of bone in order to inform specialist MDTs and identify areas in which collaboration to develop more evidence aimed at strengthening future consensus might be worthwhile. We have identified an engaged and supportive professional and patient community which actively contributed to this project and constituted a key strength of this report. Although we have attempted to collect objective data about treatment, our description of the strength of consensus is necessarily subjective. The conclusions of this paper should therefore be treated with caution and are for consideration by treating teams rather than definitive recommendations.

### The ideal patient pathway and staging

The urgent referral of patients with a suspected Ewing sarcoma of bone to a specialist centre is established practice [6]. Initial evaluation with plain radiographs and MRI of the whole involved compartment and adjacent joints is universally applied. However, we have shown variation in systemic staging with some centres performing CT of abdomen and pelvis as well as chest. There is greater variation in imaging of the skeleton with centres most often performing whole body isotope bone scan and bone marrow sampling. PET/CT was also widely offered and may be as useful as bone marrow sampling [7]. However whole body MRI scan was not routinely offered and given recent evidence about its sensitivity may be a better staging investigation [8]. Further evaluation of the optimal staging investigations for Ewing sarcoma is warranted.

### The multidisciplinary team and decision making

The treatment of patients with Ewing sarcoma requires a properly constituted MDT with expertise in medical oncology, radiotherapy, and surgery [6, 9]. We have shown there is almost universal use of MDT discussion, and adoption of a wide range of existing treatment protocols. Wide discussion outside the treating MDT, for example in a national forum or with another centre is not usual in the majority of countries, although the EICESS 92 experience indicates that a more unified approach could be explored to determine if it will improve outcomes for patients and has been adopted by the CESS group in Germany [4, 10]. The wider adoption of national or international MDT discussions has some intuitive appeal but demands further evidence.

### Timing and approaches to decisions about local treatment

Complete resection of the primary tumour continues as the preferred approach, with radiotherapy as an alternative if surgery is not feasible, unacceptably morbid or if the prognosis is poor (for example with widespread bone metastases). The majority of patients receive combined treatment. Evidence in favour of one or other treatment approach comes predominantly from retrospective studies and is difficult to interpret because there is an inherent bias in treatment selection.

#### Shared decision making

Shared decision-making involves a collaborative approach to treatment decisions based on an understanding of the patient’s priorities for their own lives: we showed strong consensus that decisions should be made together with patients and families as early as possible in the treatment pathway. However, there is no evidence that this approach is of benefit in patients with Ewing sarcoma specifically and standard mechanisms for supporting high quality decisions do not exist. There is an opportunity to develop these and to use existing outcome data to allow patients, families and treating teams to make fully informed treatment decisions [11].

#### Decisions about local treatment in the presence of metastatic disease

Patients with bone metastases at diagnosis have a poor prognosis [12], and therefore there was an expected strong consensus for avoiding morbid surgery. The treatment of multiple bone metastases with radiotherapy is likely reasonable if they are small in number and easily treatable, but there is little evidence for improved outcomes associated with this approach. The better prognosis of patients with pulmonary metastases alone supports a more aggressive approach to local treatment, including, for example, surgical resection of the pelvis. However, decisions about local treatment may be more nuanced given the lower expectation of cure.

### Pathology and molecular biology

#### Biopsy techniques and approaches

The principles of biopsy of musculoskeletal tumours are well established. An image guided needle biopsy is the most widely adopted technique, with open incisional biopsy preferred by some. The latter has the advantage of providing more material for diagnostic studies and research at the risk of greater local contamination. This may become more important as biological studies, for example whole genome sequencing become integrated into the diagnostic and treatment pathway.

Biopsy of other areas of potential involvement, such as lymph nodes or bone metastases can also be important at presentation and *before chemotherapy*, particularly if aggressive local treatment such as surgical excision might be considered. Evidence is needed to support this more aggressive approach to oligometastatic disease including the identification of patients who might benefit.

#### Assessment of response to chemotherapy

There was strong consensus that the histological response to chemotherapy was important when considering the effectiveness of local treatment, although interpretation of this is made more difficult after preoperative radiotherapy. The level at which a response is defined as adequate varies: Greater than 90% necrosis was accepted by many participants, but others only consider a complete histological response as ‘adequate’ [13]. Standardisation of radiological response parameters is also needed.

### Surgery

#### Principles of surgery for Ewing sarcoma

At presentation, Ewing sarcomas typically have a large extra-osseous mass which infiltrates local anatomical structures. Although the mass may reduce following chemotherapy, viable tumour cells may remain in these infiltrated structures. The principle that all of the structures involved at presentation should either be surgically removed or included in the radiation field has become established within the NEMDT in recent years and reached strong consensus after discussion at this meeting. This included the resection of perilesional oedema if reasonable. In practice, however, local treatment decisions are often more pragmatic, driven by an understanding of what is reasonable, the associated morbidity and the expectation of cure.

Surgical margin assessment aims to evaluate whether there is residual tumour in the local site, and there is some variation in what is believed to be an adequate surgical margin. Future assessments may need to go beyond the traditional evaluation of the resected specimen and be combined with response to chemotherapy to give a better risk stratification for local recurrence.

#### Complications of surgery

A key consideration in local treatment is the interaction between surgery and radiotherapy. Radiotherapy is associated with an increased risk of wound complications and deep implant infection [14]. Furthermore, the smaller treatment volume and potential for lower doses have increased the use of preoperative radiotherapy, particularly in the pelvis. Disadvantages include an increase in complications of surgery or that surgery may be more technically challenging if tissue planes become difficult to identify. However, the latter was not supported in the meeting.

Complications of surgery such as infection or wound failure can delay the resumption of chemotherapy. Therefore in some cases where the risk of such complications is sufficiently high, it has been suggested that surgery is delayed until the end of chemotherapy. However, no consensus was reached on this question.

### Radiotherapy

Radiotherapy is most frequently delivered with photons, although as access improves, more patients are receiving proton beam treatment. Photon radiotherapy is best delivered via a 3D-conformal CT-plan, or increasingly with intensity modulated radiotherapy, which frequently allows for superior dose delivery and normal tissue sparing. For some patients, proton beam therapy may offer an advantage in delivering the prescribed radiotherapy dose (if it is at the higher end of the dose range) or in sparing normal tissues and reducing late toxicity.

We have shown variations in the approach to radiotherapy and different approaches to the treatment of pathological fractures, lymph nodes and the indications for and timing of radiotherapy. Further evaluation of the optimal delivery of radiotherapy is required in clinical trials.

### Anatomical site variations

#### Pelvis/sacrum

About a quarter of Ewing sarcomas involve the pelvis and/or sacrum [15]. Pelvic tumours tend to be larger, with a higher risk of metastasis and poorer survival [15]. The soft tissue mass associated with a pelvic Ewing tumour can be large and may involve adjacent critical anatomical structures such as the iliac vessels, bladder and rectum. Local recurrence rates after resection of pelvic tumours can be higher than other sites [16]. Retrospective series suggest that patients treated with surgery have better overall survival [17] and that those treated with surgery and radiotherapy together have lower local recurrence rates [18], however there is great variability in preferences for local control [19, 20]. Furthermore, more recent reports have suggested definitive proton treatment can be associated with high local control rates [21].

Surgical resection of a pelvic or sacral sarcoma is routinely associated with surgical complications, long term morbidity and loss of physical function, particularly after resection of the acetabulum and major (often sacral) nerves [22, 23]. The determination of what is a resectable tumour in the pelvis depends to a large extent on the degree of morbidity acceptable to the patient and their family. There was consensus that tumours which were too morbid to resect include those which cross the midline in the sacrum and those which would require major visceral resection. These are useful guides for treating teams and demand the longer-term collection of physical functioning outcomes to inform decision making.

After preoperative radiotherapy, the expected increased risk of complications means that surgeons are less likely to recommend allograft or endoprosthetic reconstruction. After periacetabular resection, a “hanging hip” reconstruction may therefore be preferred [24].

#### Spine

Two thirds of patients with Ewing sarcoma of the spine undergo urgent decompression at presentation which complicates subsequent local management [25]. However, there was moderate consensus that a patient with spinal cord compression and neurological compromise from Ewing sarcoma should undergo urgent surgical decompression if it is indicated. Radiotherapy should then include all of the original tumour volume and other areas potentially contaminated.

For those with spinal tumours who do not have neurological compromise, it is appropriate to treat first with urgent biopsy and chemotherapy which may lead to a reduction in tumour size and avoid urgent surgical decompression.

The decision about what is resectable within the spine should be made by a surgeon with relevant site-specific experience working within a multidisciplinary team. There may be some benefit to surgical excision if the morbidity is acceptable, but there are no randomised trials and local control rates with radiotherapy alone are high [16, 26]. When surgery is undertaken consideration should be given to the type and positioning of instrumentation used in reconstruction. Traditional titanium implants may significantly impair the ability to deliver PBT and have a deleterious effect on follow-up MRI imaging.

#### Chest

The use of radiotherapy for chest wall tumours is related predominantly to pleural involvement either before or after surgery. A pleural effusion is not on its own an indication for radiotherapy [27].

#### Extremity

Ewing sarcoma of the extremity should be treated with wide resection if feasible, and in the majority limb sparing surgery is possible, with amputation rates around 8.4% [15]. Amputation is associated with the best local control rates, but usually the greatest loss of function. It should be pointed out that while surgery is certainly favoured secondary to other important considerations, overall prognosis may not be influenced by local control modality [28]. Exceptions to this rule include significant resections of the foot or tibia which would result in lower levels of physical functioning than transtibial amputation [29]. In the upper limb there is a greater emphasis on the preservation of function, and teams may rely more on adjuvant radiotherapy to achieve local control.

Reconstruction of the proximal tibia is associated with relatively high surgical complication rates, including infection [30]. Adding radiotherapy to a surgical reconstruction may increase this risk and may lead to a failed reconstruction. In this situation therefore, there was consensus in favour of preoperative radiotherapy but recognition that amputation may be a reasonable alternative for some patients.

### Local therapy in metastatic and relapsed disease

Although bone metastases are associated with a dismal outcome, there was support for a more aggressive approach using focussed radiotherapy and possibly surgery if there are only a small number of bone metastases [31]. Similarly, there was some support for an aggressive approach to potentially involved lymph nodes, including biopsy or sampling at presentation and surgical resection and/or radiotherapy as part of definitive local therapy.

After isolated local relapse, there will routinely be a question about whether further systemic treatment should be supplemented with local treatment. There was strong consensus that it was reasonable to consider even radical surgery such as hemipelvectomy in this situation, but it is important to balance the definite morbidity of the treatment with any small potential to improve the overall poor outcome, particularly in early relapse. All of these settings need further evidence to guide clinicians.

### Future research

The variation in practice recorded in this initiative highlights areas in which research and clinical trials could be usefully performed in order to develop stronger evidence-based approaches to primary tumour management. These include:

- The role of new imaging technology for more accurate staging.
- Developing methods for early and shared decision-making about local treatment.
- The role and benefit of specialist national MDT review.
- Definitions of radiological and histological responses to chemotherapy.
- Studies of type, timing and dose of radiotherapy. As an example, incorporation of prospective questions about radiotherapy delivery within the next generation of European clinical trials for Ewing sarcoma are already well advanced.

Re-examination of these areas of consensus in a further international workshop, preceded by an updated survey, would certainly be worthwhile.

## Conclusion

Achieving consensus about local therapy decisions in Ewing sarcoma is possible but several areas lacking consensus remain. There is consensus about the central role of an MDT, and the services patients require for optimal treatment. However, variation in approaches between centres have been described, the significance of which is uncertain. Areas where clinical trials may be developed have been identified.

## Supplementary information

**Additional file 1: Appendix 1.** Summary of premeeting survey questions. **Appendix 2.** Summary of cases presented.

## Data Availability

Data sharing is not applicable to this article as no datasets were generated or analysed during the current study.

## Abbreviations

CT: Computed tomography
IMRT: Intensity modulated radiotherapy
MDT: Multi-disciplinary team
MRI: Magnetic resonance imaging
NEMDT: National Ewing Multidisciplinary Team panel
PBT: Proton beam therapy
PET/CT: Positron emission tomography and computed tomography

## Footnotes

1 Due to constraints on accessing personnel with sufficient expertise, absence of adequate data and because of time constraints within the programme.
