# Supplementary material for: Seeking international consensus on approaches to primary tumour treatment in Ewing sarcoma
Source: Clin Sarcoma Res. 2020 Nov 17;10:21. doi: 10.1186/s13569-020-00144-6 (PMC7672819; doi:10.1186/s13569-020-00144-6)
Supplement: Supplementary file 1 — Additional file 1: Appendix 1. Summary of premeeting survey questions. Appendix 2. Summary of cases presented. [file 13569_2020_144_MOESM1_ESM.docx]

### Additional Materials

### Appendix 1. Summary of premeeting survey questions

**Section 1. Background information.**

1.1. Which centre and country do you work in?

1.2. Please select the professional group to which you belong: (orthopaedic surgery/clinical oncology/medical oncology/paediatric oncology/histopathology/radiology/other)

1.3. Did you attend the 1^st^ International Consensus meeting in Birmingham in 2007?

1.4. Approximately how many new patients with ES does your centre treat every year?

**Section 2. Diagnostic and staging investigations.**

2.1. At diagnosis do patients in your centre routinely have:

1. Conventional x-ray in 2 planes?
2. MRI of whole involved compartment with adjacent joints?
3. Whole body MRI?
4. CT chest only?
5. CT chest, abdomen and pelvis?
6. Whole body PET/CT?
7. Isotope bone scan?
8. Bone marrow sampling?
9. Other staging investigations (please specify)?

2.2. Do patients have their diagnostic biopsy in your bone cancer centre?

2.3. What is the standard for biopsy in your centre?

2.4. Are biopsy tracks marked so that they may be located at time of definitive procedure?

2.5. Are biopsy specimens tested for cytogenetic abnormalities, Including EWS translocation?

2.6. Do you sample lymph nodes that may be involved on imaging?

2.7. Do you biopsy suspected bone oligometastases?

2.8. Do you routinely bank tissue for research?

**Section 3. Patient pathway and services.**

3.1. Are patients in your centre routinely discussed in a multidisciplinary team (MDT) meeting to plan treatment?

3.2. Which protocols/guidelines have been adopted in your centre for patients with ES?

3.3. Which of the following (services) do your patients have access to?

3.4. If you could do one thing to improve the service you offer for patients with ES, what would it be?

3.5. Please indicate your opinion on the following statements:

1. The radiological response to neoadjuvant chemotherapy is important when considering local treatment
2. The response to chemotherapy in the resected specimen is important when considering the effectiveness of local treatment
3. An adequate response to chemotherapy should be defined as >90% necrosis
4. An adequate response to chemotherapy should be defined as 100% necrosis
5. Patients with bone metastases should have the same local treatment as those without
6. Patients with pulmonary metastases should have the same local treatment as those without
7. The surgical margin assessment is a reliable indicator of tumour left in the patient
8. An adequate surgical margin is one in which there is no viable tumour at the edge of the resection specimen
9. An adequate surgical margin is one in which all of the anatomical structures involved before chemotherapy have been completely removed

**Section 4. Surgery for ES of bone. This section is only to be completed by surgeons.**

4.1. When planning surgical resection, do you plan to remove the biopsy track?

4.2. Do you consider resecting all of the volume/anatomical structures involved BEFORE chemotherapy?

4.3. Do you consider resecting all of the volume/anatomical structures involved AFTER chemotherapy?

4.4. Which of the following reconstructive techniques do you have access to/ use as required? Please indicate all that apply.

4.5. Please indicate your opinion on the following statements:

1. Some patients at high risk of surgical complications should complete all of their chemotherapy before surgical treatment
2. Radiotherapy has a negative impact on outcomes after endoprosthetic replacement of a long bone
3. Radiotherapy has a negative impact on outcomes after allograft reconstruction
4. Preoperative radiotherapy makes surgery more difficult technically
5. Debulking surgery should be considered when a tumour cannot be completely resected.
6. Local recurrence at the primary site has an impact on overall survival
7. It is appropriate to surgically resect lymph nodes if there is suspicion of tumour involvement
8. Surgical resection of bone metastases improves survival
9. I would resect a bone metastasis if the morbidity was acceptable
10. Pelvic spacers should be considered to reduce the morbidity of radiotherapy in that site
11. Amputation leads to better outcomes than proximal tibial replacement and radiotherapy
12. I am less likely to recommend amputation in the upper extremity than the lower because of the impact on physical functioning
13. I would consider radical surgery (such as amputation or hemipelvectomy) to treat locally recurrent Ewing’s sarcoma if there are no metastases
14. Radiological response to neoadjuvant chemotherapy is a factor in determining surgical planning
15. Patients should have the opportunity to explore local treatment options as soon after diagnosis as possible
16. Decisions about local therapy should be made in collaboration with patients and families

4.6. In your opinion, what makes a pelvic tumour unresectable? Please indicate all that apply.

4.7. When would you consider amputation of a limb for a patient with an extremity ES? Please indicate all that apply.

**Section 5: Radiotherapy for ES of bone. This section is only to be completed by clinical oncologists.**

5.1. What dose do you recommend for preoperative radiotherapy?

5.2. What dose do you recommend for postoperative radiotherapy?

5.3. What dose do you recommend for radiotherapy in unresectable disease?

5.4. Should pre-operative radiotherapy be given when:

1. Tumour is close to critical anatomical structures which would be morbid to resect surgically (eg major nerve, blood vessel)?
2. A close or positive surgical margin is expected based on the PREchemotherapy scan?
3. A close or positive surgical margin is expected based on the POSTchemotherapy scan?
4. Tumour over a certain volume (please state volume)?
5. A pleural effusion related to a chest wall tumour?
6. Pleural involvement with a primary tumour?
7. Presentation with a pathological fracture?
8. A tumour in the bony pelvis?
9. A poor radiological response to neoadjuvant chemotherapy

5.5. Should post-operative radiotherapy be given when:

1. Viable tumour at the surgical margin
2. Incomplete excision of pre-chemotherapy tumour volume?
3. Poor histological response – (please give definition)
4. Tumour over a certain volume (please state volume)
5. A pleural effusion related to a chest wall tumour?
6. Pleural involvement with a primary tumour?
7. Presentation with a pathological fracture?
8. A tumour in the bony pelvis?
9. A poor radiological response to neoadjuvant chemotherapy
10. Other indications for post-operative radiotherapy (please specify)

5.6. Please indicate your opinion on the following statements:

1. Involved lymph nodes should be treated with radiotherapy
2. As proton beam treatment becomes more available, indications will expand
3. I can make a decision about radiotherapy based on the imaging of the tumour at presentation
4. Craniospinal radiotherapy is indicated for patients with epidural disease

6. Please use this space for any further comments about areas of uncertainty for local control in ES.

### Appendix 2. Summary of cases presented

**Case 1.** 9 year old male. Localised extensive proximal femur lesion including acetabulum and femoral head. Restaging scans after chemotherapy. New contralateral lesions femur and pelvis.

**Case 2.** 12 year old female. Iliac tumour with pulmonary and pleural based metastases in all lobes. Incomplete radiological response to chemotherapy. Complete resolution of pulmonary nodules,

**Case 3.** 22 year old male. Proximal tibial tumour with diffuse marrow infiltration. Incomplete radiological response to chemotherapy.

**Case 4.** 9 year old male. Proximal fibular tumour, solitary bone metastasis in spine. Fibulectomy with 100% necrosis.

**Case 5.** 22 year old female, sacro-iliac and L5 transverse process tumour, lesions in glenoid and humerus on PET-CT

**Case 6.** 24 year old female, distal fibular tumour with small FDG avid popliteal and iliac nodes.

**Case 7.** 15 year old male. Localised fibular tumour. Good response to chemotherapy.

**Case 8.** 30 year old female. Localised scapular tumour. Preoperative radiotherapy, 90% post chemotherapy necrosis, 2mm margin.

**Case 9.** 21 year old male. Femoral tumour with large extraosseous component and multiple pulmonary nodules, suboptimal response to chemotherapy. 30% viable tumour with clear margins.

**Case 10.** 12 year old male. Localised tumour of ilium, excellent response to chemotherapy.

**Case 11.** 14 year old female. Localised tumour of femoral diaphysis. Incomplete radiological response to chemotherapy.

**Case 12.** 9 year old male. Tumour extending into L4/5 and L5/S1 foramina. Very good radiological response to chemotherapy.

**Case 13.** 38 year old female. Localised L2 tumour. Good radiological response to chemotherapy. En bloc resection. 60% necrosis. Anterior margin involved.

**Case 14.** 19 year old male. Spinal cord compression from localised L2 tumour. Emergency decompressive surgery.

**Case 15.** 13 year old male. Large sacral tumour extending into ilium. Multiple small lung metastases.

**Case 16.** 15 year old female. Localised tumour of hemipelvis including iliac bone and sacral ala.

**Case 17.** 5 year old female. Localised 6^th^ rib tumour with pleural involvement, good radiological response to chemotherapy.

**Case 18.** 18 year old female. Distal humerus tumour with joint involvement, large extraosseous component. Partial radiological response to chemotherapy.
